# Automatic Regularization of TomoSAR Point Clouds for Buildings Using Neural Networks

**DOI:** 10.3390/s19173748

**Published:** 2019-08-30

**Authors:** Siyan Zhou, Yanlei Li, Fubo Zhang, Longyong Chen, Xiangxi Bu

**Affiliations:** 1 School of Electronics, Electrical and Communication Engineering, University of Chinese Academy of Sciences, Beijing 100049, China; 2 National Key Lab of Microwave Imaging Technology, Institute of Electronics, Chinese Academy of Sciences, Beijing 100190, China

**Keywords:** denoising, neural networks, regularization, 3-D point clouds, tomographic SAR

## Abstract

Tomographic SAR (TomoSAR) is a remote sensing technique that extends the conventional two-dimensional (2-D) synthetic aperture radar (SAR) imaging principle to three-dimensional (3-D) imaging. It produces 3-D point clouds with unavoidable noise that seriously deteriorates the quality of 3-D imaging and the reconstruction of buildings over urban areas. However, existing methods for TomoSAR point cloud processing notably rely on data segmentation, which influences the processing efficiency and denoising performance to a large extent. Inspired by regression analysis, in this paper, we propose an automatic method using neural networks to regularize the 3-D building structures from TomoSAR point clouds. By changing the point heights, the surface points of a building are refined. The method has commendable performance on smoothening the building surface, and keeps a precise preservation of the building structure. Due to the regression mechanism, the method works in a high automation level, which avoids data segmentation and complex parameter adjustment. The experimental results demonstrate the effectiveness of our method to denoise and regularize TomoSAR point clouds for urban buildings.

## 1. Introduction

In recent years, tomographic SAR (TomoSAR) has become one of the most competitive technologies for virtual city modeling [1], city planning and management [2], and geographical information system, etc. One of its potential applications is the three-dimensional (3-D) imaging and reconstruction of buildings and other human-made architectures over urban areas. TomoSAR retrieves the elevation information of target scatterers by combining a sequence of synthetic aperture radar (SAR) images obtained by parallel flight tracks [3]. Thus, TomoSAR allows for the scatterers to be located directly. Moreover, it can separate scatterers, even in layover areas [4], and is essential in the acquisition of 3-D imaging and the reconstruction of targets [5].

Research in this field mainly derive from TomoSAR point clouds. These point clouds are acquired by high-resolution SAR sensors [6,7]. However, due to the inherent defects of the imaging principle of TomoSAR, speckle effects and noises severely damage the quality of the 3-D point clouds. Notably, in complex urban areas, the SAR imaging of densely-distributed buildings, trees, outdoor stairs, and other human-made architectures suffers from serious layover effects. Multipath scattering can also influence the point clouds [8,9], which makes the obtained airborne TomoSAR point clouds dislocated and severely noise-corrupted. The poor quality of the point clouds increases the difficulty of subsequent 3-D imaging and the reconstruction of urban buildings [10]. Thus, the processing of TomoSAR point clouds is critical. Typically, denoising is the most common means of point cloud processing. Other widely-used methods for point cloud processing mainly include point height estimation and building modeling. Point cloud processing using machine learning is also a booming research field.

From the aspect of point cloud denoising, the well-known RANSAC algorithm [11] employs random sampling strategies to identify parametric models among outliers. It iteratively computes the plane of three randomly-selected points and chooses the plane that fits the data best. Thus, RANSAC can provide the proper detection of principal planes among point clouds. [12] used RANSAC in 3-D reconstruction from multiple views by local optimization. [13] proposed extracting planes with an improvement of RANSAC. Similarly, based on the RANSAC algorithm, the TomoSeed method [14] led this plane fitting method to process TomoSAR point clouds. The region growing [15,16] was also an inspiration to this method. TomoSeed first selects seeds and retrieves planar patches according to the spatial connectivity of the scatterers. With an iteration, the algorithm finally finds the optimal parameters, so that TomoSeed can reconstruct the building planes. Compared to previous works, TomoSeed largely improved the denoising performance; however, both RANSAC and TomoSeed requires that the data is segmented before plane fitting. In other words, to reconstruct a regular building, they have to separate the building roof, facade, and surrounding ground first, which significantly limits the efficiency and automation level of these two algorithms. Furthermore, the processing performance heavily relies on the selection of the parameters.

Scatterers with stray heights can directly influence the surface smoothness. Thus, methods of height estimation are of great concern when processing TomoSAR point clouds. References [17,18] processed the point clouds efficiently by estimating the heights of the principal building planes and segmented the data into several parts and did the plane fitting of the roof and ground by part. The processing performance depends on the segmentation of the raw data and the precision of the plane fitting to a large extent. Noise-corrupted urban areas may have reduced estimation precision. Thus, data segmentation should be avoided, and a plane fitting procedure should be developed so that the automation level of the methods can be improved.

Apart from the methods for point cloud denoising and point height estimation above-mentioned, 3-D building modeling is another method of point cloud processing. Reference [19] proposed the projection of 3-D TomoSAR point clouds onto the two-dimensional (2-D) ground plane. Then, the method reconstructs the 2-D building shapes by refining the footprints. The footprints are regularized according to some particular shapes, for example, the L-shape. On this basis, many other methods of building modeling have been proposed [20,21,22,23,24,25,26]. They mainly used similar projection method firstly and reconstructed the 2-D building model on the ground plane. Whereas, the authors also pointed out that most of these algorithms were only applicable to the architectures of specific shapes. Large-scale urban areas with different kinds of buildings may be difficult for current building modeling methods to handle. Furthermore, some modeling methods require a lot of prior knowledge of the buildings; however, it is sometimes difficult to obtain this prior knowledge of the target buildings, especially in most practical instances where the detected area is unknown.

In recent years, as big data analysis is becoming a fast-growing trend, research on machine learning have been widely developed. 3-D point cloud processing methods that use relative machine learning tools (e.g., convolutional neural networks) have also drawn considerable attention [27,28]. Multi-view convolutional neural network (MVCNN) [29] tried to classify the 3-D point clouds or 3-D object shapes by applying 2-D convolutional neural networks (CNNs) on multi-view images of one object, but still used the processing method of 2-D images. In contrast, some feature-based CNNs [30,31] converted the 3-D data to a vector, then used a fully connected network to undertake feature extraction and shape classification. Other methods use spectral CNNs on meshes [32,33]. VoxNet [34] and Volumetric CNNs [35,36] were pioneers that extended CNNs to the third dimension and directly employed 3-D CNNs on voxelized shapes of objects, just like using 2-D CNNs on pixeled images. Apart from this, PointNet [37,38], and its improved method PointNet++ [39], proposed a deep neural network for 3-D point cloud recognition with better performance.

In general, current methods using machine learning have mainly been designed for small indoor objects such as lamps, laptops, and furniture. However, in the case of buildings over large-scale urban areas, these methods might be relatively inefficient. The massive computation cost of convolutions and the multi-layer structures make them inapplicable. Moreover, the machine learning methods above-mentioned mainly consider the point clouds from Light Detection and Ranging (LiDAR). For TomoSAR, the characteristics are quite different. Last, but not least, current machine learning methods only aim for the 3-D shape recognition and classification of objects. However, for the practical application demand in the remote sensing field, the recognition and classification of target shapes are far from sufficient. Thus, it is unrealistic to apply current machine learning methods directly to the processing of building point clouds from TomoSAR.

Lately, an improved generative adversarial network (GAN) has been proposed to generate continuous 2-D building footprints based on SAR images [40]. This method could achieve the completion of the building footprints automatically, however, since this method returns to 2-D image processing, we believe that the detailed 3-D structural information of buildings cannot be obtained through this method.

**Figure 1:**
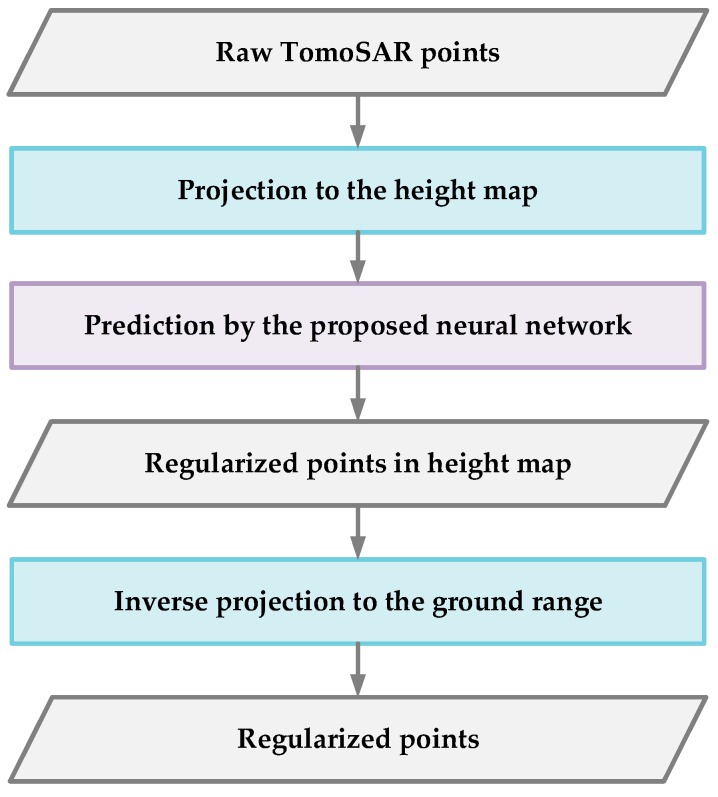
The processing flow of our regularization method for the TomoSAR point clouds.

In this paper, we propose a novel processing framework to regularize the raw TomoSAR point clouds of urban buildings with an automatic 3-D regression algorithm. The processing procedure is illustrated in Figure 1. The most arresting point of our method lies in the proposal of the neural network. By using a simple-structured, but well-designed neural network architecture, our method refines the noise-corrupted point clouds according to the original building model structures. This not only allows the smoothening of the 3-D building surface scatterers, but also provides precise preservation for the building structure. Moreover, by combining regression analysis, the proposed method avoids data segmentation and complex parameter adjustment. This improves the automation level of the point cloud processing to a large extent. The high processing efficiency of our proposed method is also noteworthy. 

The rest of this paper is structured as follows. In Section 2, we first introduce the principle of SAR tomography and give a detailed description of the processing procedure of our method. Then, we explain the projection method in detail and concretely analyze the regularization principle with the proposed neural network. In Section 3, we present a series of experimental results on both the simulated data and experimental TomoSAR data. The comparison of the results of our method, RANSAC, and TomoSeed clearly demonstrate the effectiveness of the proposed method. Section 4 furtherly analyzes the experimental results, and discusses the features and limitations of our method. In the last section, we briefly conclude this paper and provide some research directions for our future work.

## 2. Methods

In this section, the methodology of this paper is given. We first present a brief introduction of the principle of tomographic SAR. Then, we introduce the whole processing framework and provide a detailed description of each processing step in succession. It must be clarified that the method of projection and inverse projection used in this paper (introduced in Section 2.3) is a slight improvement based on that in [8]. In contrast, we proposed directly projecting by every point rather than by per resolution cell. This improvement guarantees the resolution of the projection result. 

Furthermore, what is most noteworthy is the 3-D surface regression method proposed in our work (presented in Section 2.4) and is the first trial of the regression algorithm on TomoSAR point clouds of buildings. Via the neural network, our method provides excellent regularization performance for those noise-corrupted points automatically and is the main innovation of our work.

### 2.1. SAR Tomography

**Figure 2:**
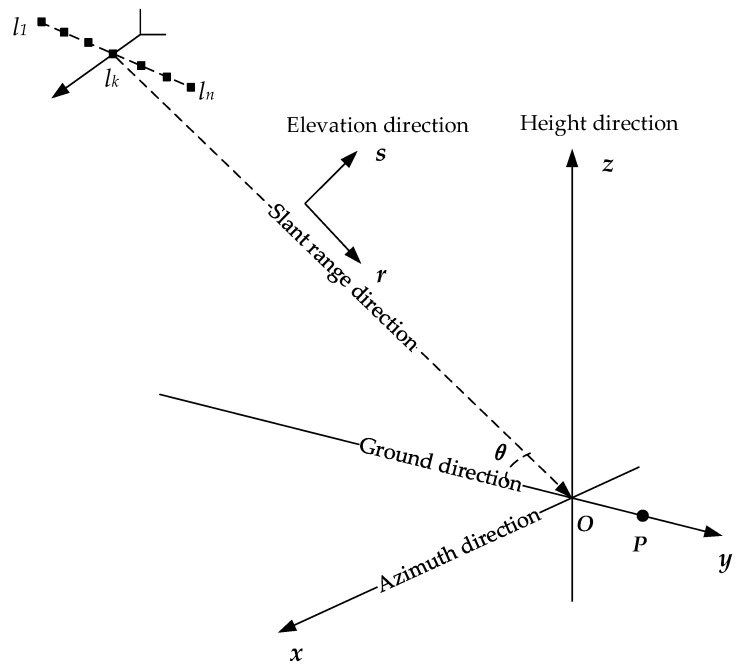
Schematic geometry of tomographic SAR (TomoSAR).

Tomographic SAR, or TomoSAR for short, extends the 2-D imaging principle of SAR to the elevation dimension. It exploits the acquisitions from different angles to reconstruct the elevation reflectivity for every azimuth-range cell [5]. Thus, TomoSAR allows the location of point targets in the 3-D space. The 3-D imaging of the point targets in the target area also becomes realistic by using TomoSAR. In our work, we mainly focused on airborne TomoSAR. Figure 2 gives the geometry of the imaging principle of airborne TomoSAR.

For every point target, there is one cylindrical coordinate (r,s,x) and one rectangular coordinate (x,y,z), respectively, for example, for target *P*, its cylindrical coordinate is $\left(r_{P},0,0\right)$ and its rectangular coordinate is $\left(0,y_{P},0\right)$. The *k*th radar sensor has the corresponding rectangular coordinate $\left(0,r\cos\theta +l_{k},r\sin\theta \right)$. Thus, the slant range between target *P* and the *k*th radar sensor can be represented as

(1) $$R(l_{k})=\sqrt{{\left(r\cos\theta +l_{k}-y_{P}\right)}^{2}+{\left(r\sin\theta \right)}^{2}}$$

Assuming in the far field condition that we have $r\gg l_{k}$ and $r\gg y_{P}$, with the Taylor expansion, Expression (1) can be written as

(2) $$\begin{array}{cc} R(l_{k}) & \approx r-y_{P}\cos\theta +l_{k}\sin\theta +\frac{{\left(y_{p}-l_{k}\right)}^{2}\sin^{2}\theta }{2r}\\ & \approx (r-y_{P}\cos\theta )-\frac{y_{P}l_{k}\sin^{2}\theta }{r} \end{array}$$

Here, the first term denotes the ground range position, and the second term is related to the elevation position. Thus, the ground range and the elevation of target P can be obtained. Therefore, TomoSAR allows the location of targets in 3-D space and provides a prerequisite for our subsequent analysis.

### 2.2. Processing Procedure

**Figure 3:**
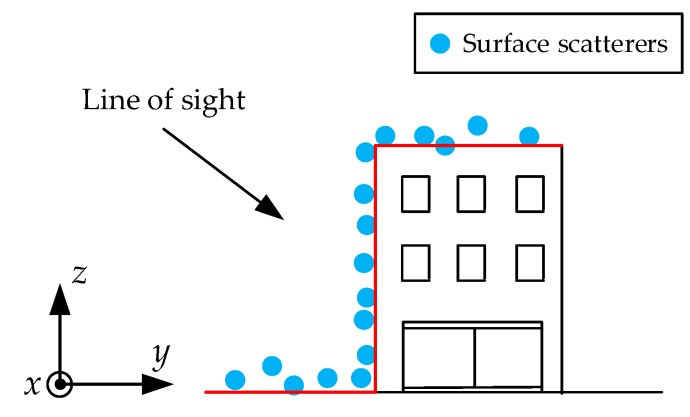
Schematic geometry of the 3-D building model. Red lines denote the “visible” plane.

For simplicity, in the following analysis, we assumed that the input point clouds of our method were projected from the slant range domain to the ground range domain by using the method described in [18]. The schematic geometry of one single target building is illustrated in Figure 3. Considering the side-look imaging principle of TomoSAR, we made a simplifying assumption that the radar did not penetrate the building and ground, so that the surface scatterers can be “seen” only from one side of the building [18]. Then, we directly used these surface scatterers to represent the building structure.

Our regularization method takes the raw TomoSAR point clouds of buildings as the input and output a bunch of refined points, where the outputted points can represent the building surface well. With the assumptions above-mentioned, the regularization of point clouds can be conducted with the following procedure (as depicted in Figure 1). First, the raw TomoSAR point clouds of a building (with its surrounding ground) are projected from the ground range domain to the height map. This step is concretely introduced in Section 2.3. Afterward, for these projected scatterers in the height map, the proposed neural network gives their prediction heights one by one. Thus, the scatterers are regularized to new positions with the predicted heights and form a new smooth 3-D building surface along with the ground truth. This process step is described in Section 2.4. Finally, by exploiting another projection, these regularized points are projected back to the ground range domain. Since the last step is an inverse process of the first step, where a brief discussion on this is provided at the end of Section 2.3.

**Figure 4:**
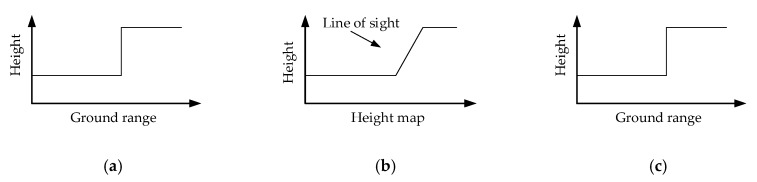
Surface changing chain. (**a**) Initial TomoSAR surface of a building in the ground range domain, which serves as our input. (**b**) Projected surface in the height map. (**c**) The final output surface of our method in the ground range domain.

Figure 4 illustrates the changing progress of the building surface in our method and mainly presents the change before and after each projection. It can be observed that the building surface experienced three conditions: from an erect “z” to a slanting “z,” and back to an erect “z.” For simplicity, here we omit drawing the building points. Points were resampled from the building surface, so they appear to have the same trend as the surface.

With the processing procedure above-mentioned, the regularization of 3-D building point clouds can be achieved automatically. In the following sections, we provide detailed introductions on each step of our method. The following analysis obeys the same assumptions given at the beginning of this section.

### 2.3. Projection

As depicted in Figure 3, each point of the building and surrounding ground can be represented by a Cartesian coordinate (*x*, *y*, *z*). Here, *x* denotes the azimuth, *y* denotes the ground range, and *z* denotes the height, respectively.

**Figure 5:**
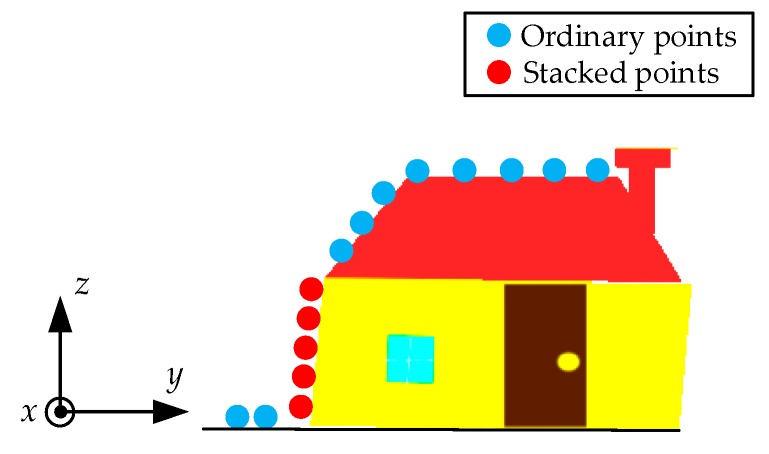
Schematic geometry of the data stack on the building facade.

With the side-look imaging principle of TomoSAR, for a regular building, its facade is composed of several surface scatterers from one side. From Figure 5, we can observe that the points are piled up along the facade, in other words, one given azimuth-ground range (*x*, *y*) corresponds to more than one scatterer. Thus, one azimuth-ground range (*x*, *y*) corresponds to more than one height value. This data stack is harmful for the subsequent height prediction (in Section 2.4). During training, the algorithm would mistakenly take all these height values of one azimuth-ground range to predict, so the convergence may be led toward the wrong optimization direction. Thus, it is first necessary to distinguish these points and solve the data stack problem.

**Figure 6:**
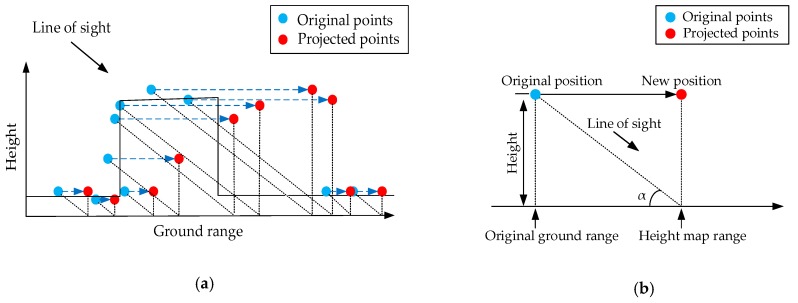
Projection diagram. (**a**) Transformation progress of the surface scatterers. Black dotted lines denote the triangle relationship, and blue dotted lines denote the change before and after the projection. (**b**) The triangle relationship between the look-down angle and the original point height.

Considering the principles of TomoSAR, the sensors collect the data from one side. We assumed that the sensors would not penetrate the architectural surface. Along the line of sight direction, every azimuth-ground range would correspond to only one scatterer [8]. Thus, surface scatterers are distinguishable when seen from the line of sight direction. Therefore, to solve the data stack problem, we employed a projection [8] on the building surface scatterers along the line of sight direction. The projection procedure is as follows:

1. First, the height map ranges of scatterers are computed according to the triangle relationship between the look-down angle and the original scatterer heights (see Figure 6b). The height map range can be computed with:
  (3) $$y^{\prime }=y+z\tan\alpha$$
  where y is the original ground range of the scatterer; z is the original height of the scatterer; α is the look-down angle; and $y^{\prime }$ is the projected height map range of the scatterer. The azimuth x is unchanged in this projection.
2. Then, keeping the heights unchanged, the scatterers are converted to the height map with the calculated height map ranges. Thus, the height map coordinate of every scatterer becomes $\left(x,y^{\prime },z\right)$.

Figure 6a illustrates the transformation progress of the projection.

With the projection method above-mentioned, the surface scatterers are now distinguishable in the height map. One azimuth-height map range coordinate corresponds to only one scatterer, so the data stack problem along the facade is solved. 

**Figure 7:**
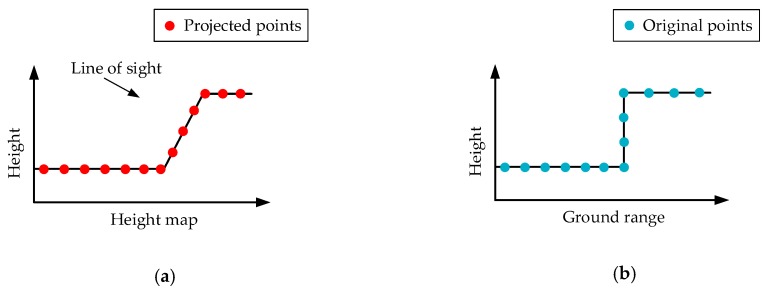
Schematic geometry of the building structure. (**a**) Rectified building shape in the height map. (**b**) Original building shape in the ground range.

Then, as seen in Section 2.4, these projected points in this height map serve as the input of the neural network. The network then carries out the height prediction to every surface scatterer, so the scatterers are regularized along the building surface by changing their heights. As depicted in Figure 7a, the building is now a distorted z-type structure with a slanting facade. The line of sight is perpendicular to the slanting facade. Figure 7b illustrates that the original building is an erect “z” with a perpendicular facade. To keep the building shape unchanged, we must rectify the whole building structure into its original shape (see Figure 7b).

**Figure 8:**
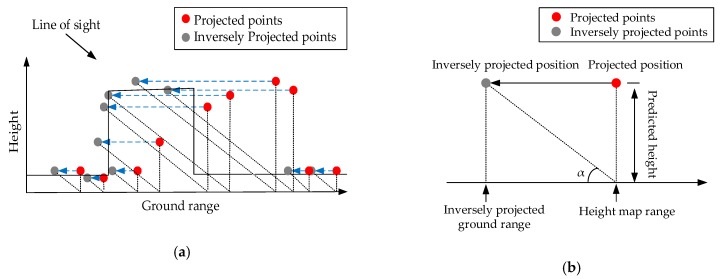
Inverse projection diagram. (**a**) Inverse transformation process of the surface scatterers. Black dotted lines denote the triangle relationship, and blue dotted lines denote the change before and after the inverse projection. (**b**) The triangle relationship between the look-down angle and the predicted point height.

Thus, we employed an inverse projection on the regularized points (see Figure 8a). This inverse projection is quite similar to the projection above-mentioned. It exploits the triangle relationship between the predicted heights and the look-down angle, which is depicted in Figure 8b, to project the scatterers back into the ground range domain. The inverse projection procedure can be explained as follows:

(1) First, the new ground ranges of scatterers are computed according to the triangle relationship between the look-down angle and the predicted heights. The height map range can be computed with:
  (4) $$y^{\prime \prime }=y^{\prime }-z^{\prime }\tan\alpha$$
  where $y^{\prime }$ is the projected height map range of the scatterer; $z^{\prime }$ is the predicted height of the scatterer; α is the look-down angle; and $y^{\prime \prime }$ is the inversely projected ground range. The azimuth x is still unchanged in this projection.
(2) Then, keeping the predicted heights unchanged, the scatterers are converted to the height map with the calculated ground ranges. Thus, the height map coordinate of every scatterer becomes $\left(x,y^{\prime \prime },z^{\prime }\right)$.

Thus, the refined points in the height map are projected back to the ground range domain and serve as our final output.

### 2.4. Neural Network Architecture

For high-quality 3-D building reconstruction, the smoothness of the building surface is essential. The heights of the scatterers need to be estimated accurately since they directly influence the surface smoothness.

In previous works, the scatterer heights have been estimated by the plane fittings of the facade, roof, and ground, respectively. Thus, the segmentation of building structures was required, which inevitably deteriorated the precision and efficiency of the processing. In this regard, we proposed a 3-D surface regression of the whole building structure at one time.

**Figure 9:**
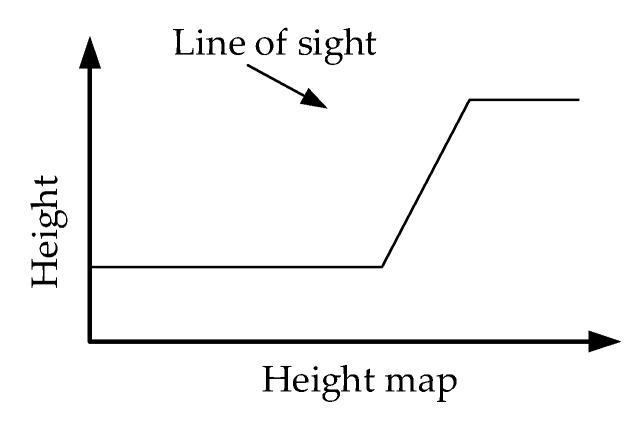
Schematic geometry of the building surface in the height map.

In the following analysis, we assumed that the scatterers were projected to the height map using the projection above-mentioned. Thus, the scatterers can be denoted by a new 3-D coordinate set (***x***, ***y***, ***z***). Inspired by regression analysis, we aimed to determine the relationship between the azimuth-range and the scatterer height, namely, the 2-D coordinate set (***x***, ***y***) and the original height vector ***z***, where ***x*** is the azimuth vector, and ***y*** is the ground range vector. Due to the projection above, the current building structure is like a distorted “*z*” and has a relatively high complexity (see Figure 9). Unlike ordinary linear regression, in this case, it is hard to find a specific functional expression to represent the structure. Using a piecewise linear function to represent the structure could be an ordinary solution. However, piecewise function requires dividing the data into several parts and undertake the linear regression by part. This would result in low processing precision and low working efficiency. Thus, avoiding data segmentation and processing the data at one time is critical.

In this regard, we propose a novel neural network architecture to solve the problem automatically. With the multilayer structure and the trained parameters, a well-designed neural network can extract the global features of the complex data and process all the data at one time as well as avoid segmenting the data.

**Figure 10:**
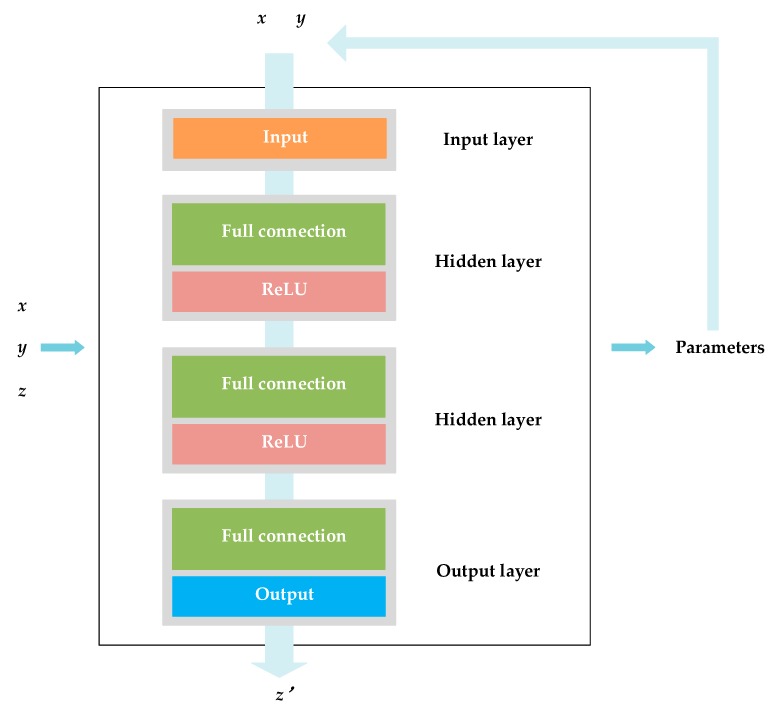
The proposed neural network architecture.

**Figure 11:**
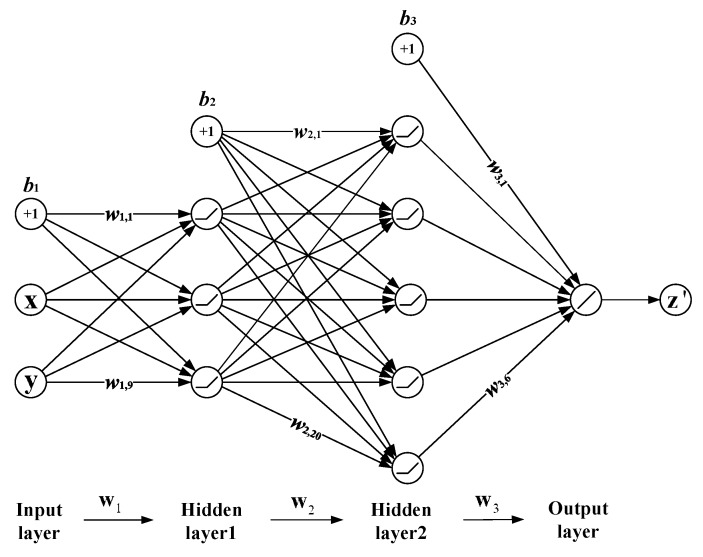
The detailed network structure.

The training progress is illustrated in Figure 10, and the proposed neural network is structured as Figure 11 depicts. It is a four-layer neural network. The first layer is an input layer where the input (***x***, ***y***) is sent into the network. The second layer and the third layer are hidden layers. Both of the hidden layers are fully connected. Here, the weights and biases are trained to represent the complex z-type building structure. For quantitative expression, the z-type structure is denoted by the functional relationship between the input azimuth-ranges (***x***, ***y***) and the output heights ***z***′. For simplicity, in the following analysis, we called the relationship between the input azimuth-ranges and the output heights as the input–output relationship for short. The ReLU function was used to activate the data. The fourth layer is a full connected output layer, where the predicted height ***z***′ is outputted. The symbol 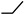 represents the ReLU function, the symbol 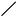 is a summation, and 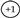 is a bias point. Through training, the weights and biases were obtained, so the network model was determined. This trained net can present the distorted z-type building structure. Then, via the trained neural network, the predicted point heights ***z***′ can be acquired by a regression. With the azimuth-ranges (***x***, ***y***) as the input, the network gives the predicted heights to the corresponding scatterers by using the computed net parameters. 

As depicted in Figure 11, *W*_1_ is a 3 × 3 matrix composed of nine weights (*w*_1,1_, *w*_1,2_, …*w*_1,9_); *W*_2_ is a 5 × 4 matrix composed of 20 weights (*w*_2,1_, *w*_2,2_, …*w*_2,20_); and *W*_3_ is a 1 × 6 matrix composed of six weights (*w*_3,1_, *w*_3,2_, …*w*_3,6_), where *w*_*i*,*j*_ is the *j*th weight in the *i*th layer. Thus, according to the network structure, the relationship between ***z***′ and (***x***, ***y***) can be written as

(5) $${z_{i}}^{\prime }=W_{3}\times \left[\begin{array}{c} Relu(W_{2}\times \left[\begin{array}{c} Relu(W_{1}\times \left[\begin{array}{c} x_{i}\\ y_{i}\\ b_{1} \end{array}\right])\\ b_{2} \end{array}\right])\\ b_{3} \end{array}\right]$$

In the following analysis, *W*_1_, *W*_2_, and *W*_3_ are collectively called *w*, and *b*_1_, *b*_2_, and *b*_3_ are collectively called *b.* Understandably, the net parameters can directly determine the net model and influence the expression of the input–output relationship. Thus, the parameters could indirectly influence the training effect. The precision of the height prediction is also related to the parameters. Therefore, it is critical to find the optimal weights and biases that can better extract the data features. 

To evaluate the precision of the height prediction, we denoted the prediction loss as ***E***. This is defined was the mean absolute estimation (MAE) of the prediction deviation (***z***′ − ***z***). The prediction loss can be expressed as

(6) $$E=\frac{1}{N}\sum_{i=1}^{N}\left|{z_{i}}^{\prime }-z_{i}\right|,$$

where $z_{i}$ is the original height of the *i*th scatterer; ${z_{i}}^{\prime }$ is the predicted height of the *i*th scatterer; and *N* is the total number of scatterers. The prediction loss represents the average deviation between the original heights and the predicted heights. It is easy to understand that the smaller the deviation becomes, the higher the prediction precision gets. Thus, the optimal parameters are the ones that minimize the prediction loss, which can be expressed as

(7) $$\underset{w,b}{\min}\frac{1}{N}\sum_{i=1}^{N}\left|{z_{i}}^{\prime }-z_{i}\right|,$$

**Figure 12:**
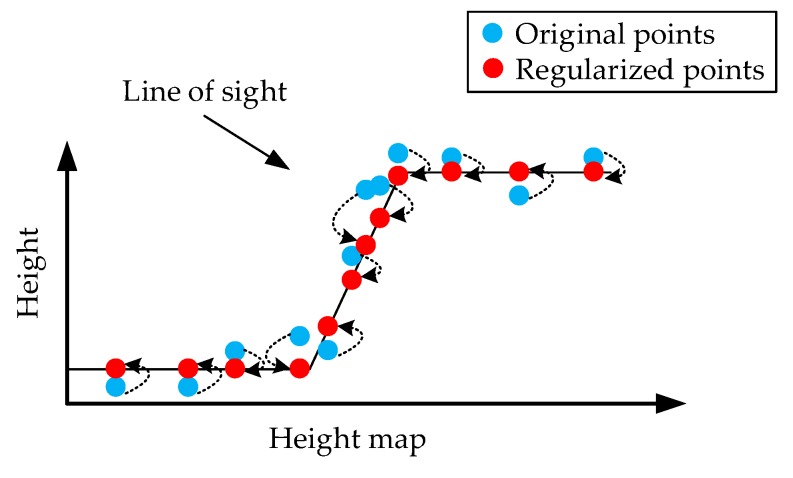
The regularization process of the points in the height map.

The parameters can be computed with the convergence of the algorithm. One of the most commonly used algorithms is the gradient descent (GD) algorithm. To improve the speed of convergence, here, we chose the adaptive moment estimation (Adam) optimization algorithm [41] to compute the network parameters. This allows for a rapid global convergence of the prediction loss. With the optimal net parameters, the predicted height vector ***z***′ can be calculated using the proposed neural network. Thus, the points are regularized and converted to new positions with coordinates (***x***, ***y***, ***z***′) (see Figure 12). It can be observed that these regularized points are smoothly distributed along with the distorted z-type building structure. They make up a new smooth 3-D building surface in the height map. This progress is a 3-D surface regression. 

In conclusion, it is a 3-D surface fitting procedure using regression analysis and neural networks. The proposed neural network is designed to represent the complex z-type building structure. The network parameters, namely, the weights and biases, provide a quantitative expression for this z-type structure. Combining the neural network with the Adam optimizer, our method can quickly and automatically find the optimal parameters. Then, the prediction heights can be given to the scatterers. Through these means, the points are regularized and make up a clean building surface. The new surface presents an excellent smoothness of the building structure.

Afterward, using the inverse projection method above-mentioned, these regularized points are projected from the height map back to the ground range domain. This processing procedure is described at the end of Section 2.3. The final output of our method is a 3-D regression result of the original building structure.

## 3. Results

In this section, we conduct a series of experiments on both the simulated data and experimental data of a real scene in China, and compare the results of our proposed method, RANSAC, and TomoSeed.

### 3.1. Simulation Data

We simulated a bunch of 3-D point clouds as if they were resampled from a single isolated building structure. The simulated building was composed of a facade, a roof, and its ground surrounding. To assess the robustness of our algorithm against errors, we added white Gaussian noise to the data.

#### 3.1.1. Single Building Facade

**Figure 13:**
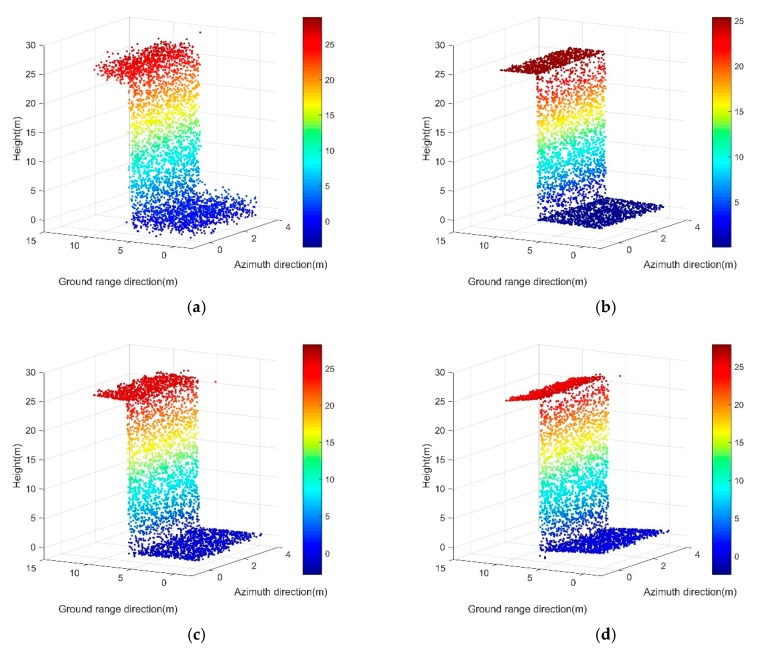
Results on the front side of the simulated building. (**a**) Original simulated point clouds from one side looking. (**b**) Results of our proposed method; (**c**) Results of the RANSAC algorithm; (**d**) Results of the TomoSeed algorithm.

We compared our method, RANSAC, and TomoSeed on 10,000 points that were just like the samples of the single isolated building facade. The input data and the experiment results are illustrated in Figure 13. 

From the comparative experiments, we observed that all three methods more or less achieved the denoising of the point clouds. The main building structure was preserved roughly. However, it can be observed in Figure 13c that some noises above the roof still existed, and in Figure 13d, the roof and ground planes were slanted. In contrast, the proposed method allowed for effective smoothening over uneven areas in the original data. The method also maintained the ground truth condition of planes and corners proximately.

**Figure 14:**
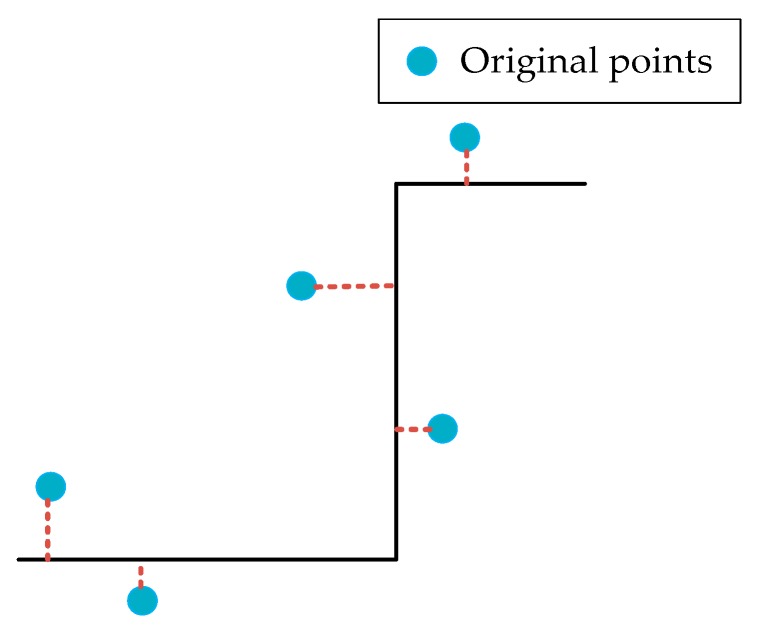
The distance computation of points. Red dotted lines denote the corresponding distances.

Since the point clouds were resampled from a simulated building facade, the ground truth of the data were known. For quantitative evaluation, we defined the regularization precision to be the deviation from the regularization result to the ground truth. For every scatterer in the point cloud, we computed its absolute distance to the nearest ground truth surface [18]. The distance is illustrated in Figure 14. The mean value of these distances is relative to regularization precision. It is understandable that the smaller the deviation, the higher the result precision. Thus, a lower precision value indicates better performance.

Table 1 gives the statistic comparison of the regularization precision and time cost of our method, RANSAC, and TomoSeed. The results indicate that our method offers the most proximate regularization to the simulated building facade truth while consuming the shortest time among the three methods.

#### 3.1.2. Single Building Corner

**Figure 15:**
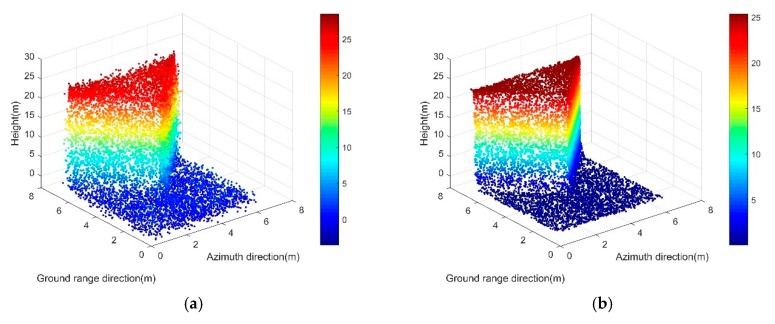

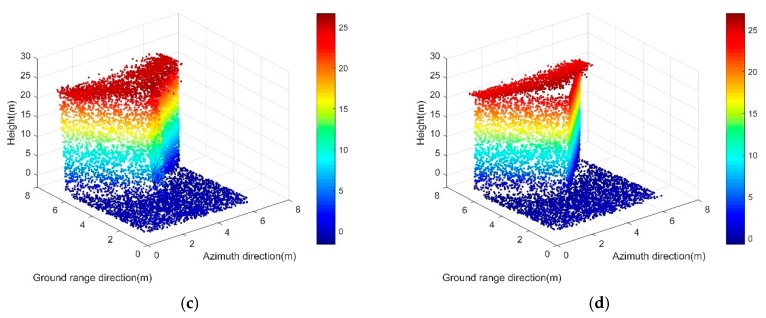
Results on the simulated data of the building corner. (**a**) Original simulated point clouds from one side looking. (**b**) Results of our proposed method; (**c**) Results of the RANSAC algorithm; (**d**) Results of the TomoSeed algorithm.

We also tested our method on the building corner data. Similarly, we simulated 10,000 points as if they were resampled from a single building corner obtained by a SAR sensor. Figure 15 gives a comparison of the input noise-corrupted data and the experimental results of our method, RANSAC, and TomoSeed, respectively.

The experimental results showed that our method demonstrated a superior performance among all three methods on the single building corner data. Unlike RANSAC and TomoSeed, there was no dislocation of the planes or shape deformation in our result. Our method allowed us to preserve the sharp edges and turnings of the corner successfully. Moreover, the method showed excellent robustness against noise and allowed us to regularize the noise-corrupted scatterers to the positions near the ground truth of the building. 

Here, we also provide a quantitative assessment of regularization precision and time cost (see Table 2). It can be observed that our method still worked better under this circumstance. The high precision and low time cost manifest as the advantages of our method. The method improved the processing precision while it only cost half the time of RANSAC. In contrast, in terms of regularization precision and time consumption, both RANSAC and TomoSeed did not perform as well. The result was within our expectation because, in this complicated situation, each step (data segmentation, plane fitting, and plane splicing) of RANSAC and TomoSeed can meet with more errors and take a much longer time.

### 3.2. Experimental TomoSAR Data

To further validate our regularization method, we tested it on the experimental point clouds. Our airborne single-pass multi-baseline InSAR system generated the experimental data. The radar system was built by IECAS in 2014. In our experiment, the radar system was installed on a Y-12 aircraft and worked at 15 GHz (Ku-band) with eight-channels in the cross-track direction. We first show the experiment results on the TomoSAR data of two single buildings.

(1) Single Building 1

**Figure 16:**
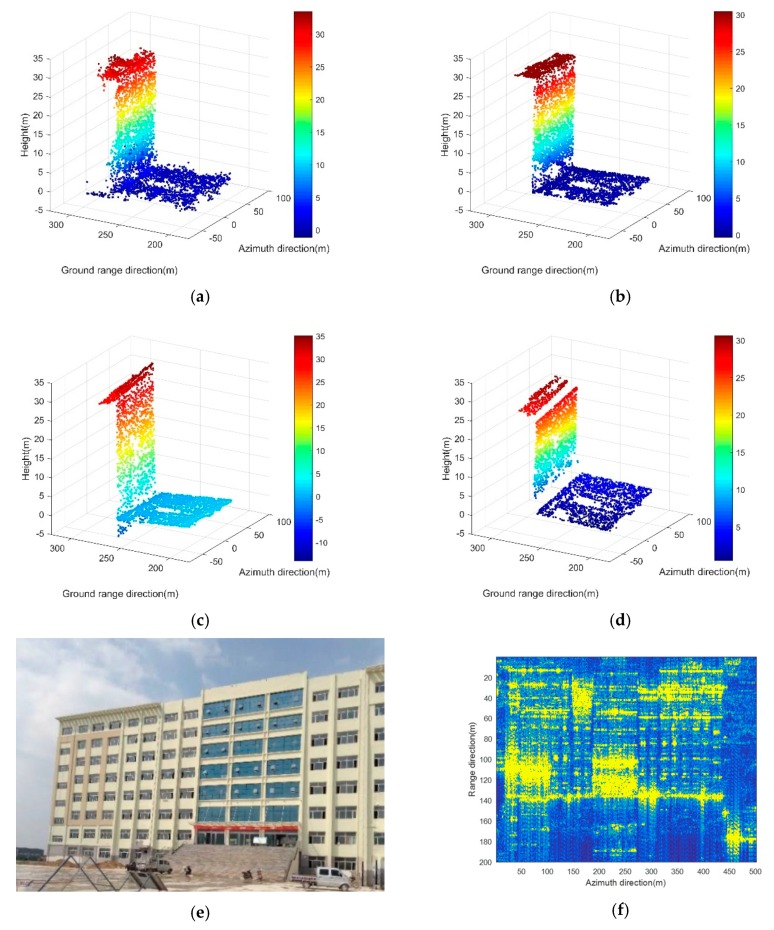
Results on the experimental TomoSAR data. (**a**) Original point clouds of the test building from one side looking. (**b**) Results of our proposed method; (**c**) Results of the RANSAC algorithm; (**d**) Results of the TomoSeed algorithm. (**e**) An optical image of the test area. (**f**) The SAR image of the test area with the Ku band.

The test area contained one single eight-story building with its surrounding ground in the city of Yuncheng in Shanxi Province, China. The base angles of the buildings were parallel to the track direction. Figure 16 gives the input data and the experimental results of our method, RANSAC, TomoSeed as well as a SAR image and an optical image of the test area as a reference. 

It can be observed that, compared with the results of RANSAC and TomoSeed, our method has a better regularization performance. The advantages of our method can be described mainly from two aspects:

(1) The planes in the results of the RANSAC and TomoSeed are dislocated. Big intervals lie among the roof, facade, and ground. In contrast, our method allowed for relatively smoother and more continuous corners;
(2) In the TomoSeed results, the three planes (the roof, facade, and ground) were slanted. In the RANSAC results, the ground rises and the facade were mistakenly lengthened. In contrast, our method kept a more precise preservation of the building structure.

For further quantitative assessment, we also recorded the time cost of the three methods (see Table 3). The experimental results indicate that our proposed method took the least computation time (only one-third of the time cost of the RANSAC). Here, we could not provide a comparison of the regularization precision. This is because in the real test area, the ground truth of the building is unknown.

(2) Single Building 2

**Figure 17:**
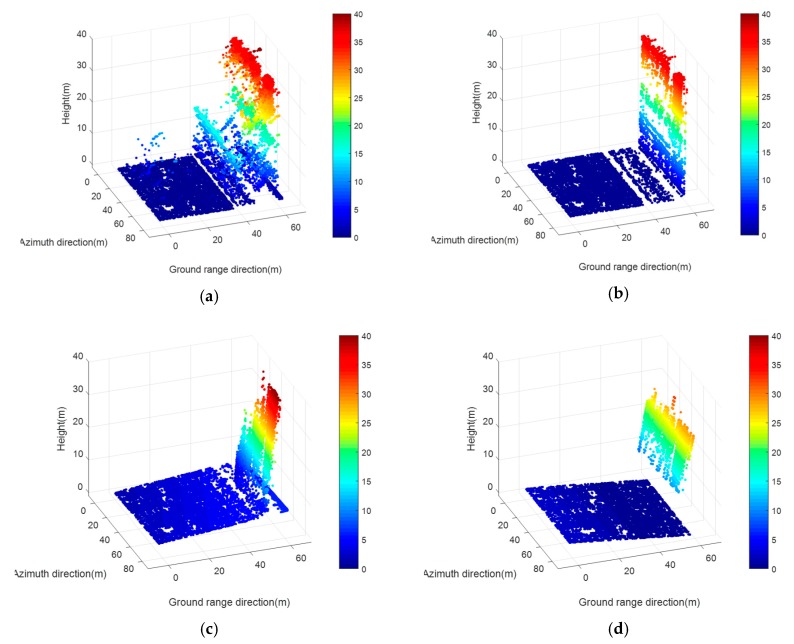

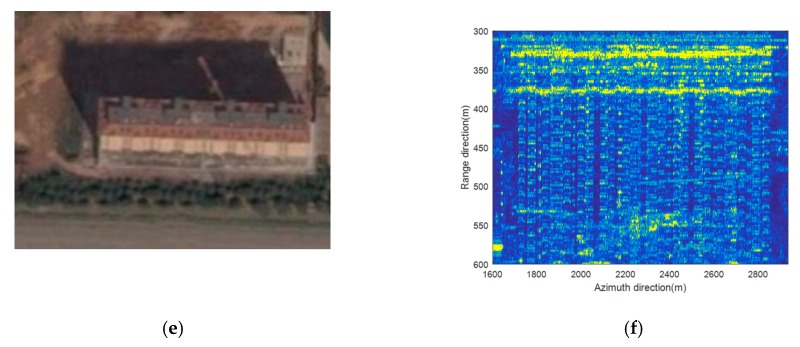
Results on the experimental TomoSAR data. (**a**) Original point clouds of the test building from one side looking; (**b**) Results of our proposed method; (**c**) Results of the RANSAC algorithm; (**d**) Results of the TomoSeed algorithm. (**e**) An optical image of the test area. (**f**) The SAR image of the test area with the Ku band.

To further evaluate our method, we tested it on another single building of 28,300 points. The data were obtained from the city of Yuncheng, Shanxi Province, China. The base angles of the buildings were parallel to the track direction. Figure 17 gives the input data and the experimental results of our method, RANSAC, TomoSeed, and an optical image of the test area as a reference.

It can be observed that the results of RANSAC and TomoSeed suffered from plane dislocations and tilts, which deteriorated the fitting performance. On the contrary, our method kept the building structure much more precisely. Meanwhile, our method had good denoising performance and effectively regularized the massive noise caused by multipath scattering into the building surfaces. 

Then, we recorded the time cost of the three methods (see Table 4). The experimental results show the high regularization efficiency of our method. 

## 4. Discussion

In this study, we investigated the current research on TomoSAR point cloud denoising and the 3-D reconstruction of building structures. We found that there existed some common shortcomings among the current point processing methods (i.e., the lack of automation and the low processing efficiency). In this regard, we proposed an automatic regularization approach based on a specially designed neural network. The method could refine the TomoSAR point clouds along with the ground truth of buildings over urban areas. 

The experimental results in Section 3 manifest the effectiveness of our method. Compared with the famous RANSAC algorithm and TomoSeed algorithm, the method in this paper has three-fold advantages:

(1) The most considerable significance of our method is its high automation level. The proposed neural network helps to represent the relationship between the azimuth-ground ranges and the heights of the scatterers. Thus, it allows for the improvement of the automation level of the extraction of 3-D building structures. In comparison, both RANSAC and TomoSeed need to segment the building data first and undertake plane fitting by part. They cannot process all the data at one time. Furthermore, our method does not rely on complex parameter adjustment or prior knowledge of the target buildings. Once our network structure is determined, the parameters can be computed automatically.
(2) The high regularization precision of our method is also noteworthy. As the method does 3-D surface regression, it allows for the extraction of global features of the building structures approximately and guarantees the robustness of our method against local noise. In contrast, both RANSAC and TomoSeed have to carry out plane fitting for every separated part. This mechanism determines the loss of global features. Thus, their results tend to be influenced by local noise, so the planes tend to be dislocated, slanting, or out of size.
(3) Last, but not least, our method is more time-saving when compared to RANSAC and TomoSeed. With the help of the Adam optimization algorithm, our method converged to the optimum solution rapidly so that the prediction loss could be minimized quickly. Thus, the 3-D regularization of urban buildings can be achieved within a short time. However, the plane fitting procedure in RANSAC and TomoSeed is not optimized, so it is very time-consuming. Moreover, as our method does not need to separate the data, we saved time in data segmentation and additionally adjusting the manual parameters.

To conclude, we achieved the automatic regularization of 3-D building point clouds from TomoSAR by using neural networks. The experimental results demonstrate that our method performed well in the 3-D regularization of buildings and the denoising of point clouds. In different scenes over large-scale areas, the method could still maintain excellent performance. Additionally, the method does not require manual data segmentation or prior knowledge of the data. The high automation level and high working efficiency make it more competitive in real-time applications as well as provide technical support for the subsequent 3-D imaging and reconstruction over urban areas. However, for buildings with complex shapes (e.g., domes), our method might not work as ideally. Fortunately, we can solve this problem to properly adjust the network structure.

## 5. Conclusions

In this paper, we proposed a novel automatic method of regularization and denoising for TomoSAR point clouds of urban buildings. Inspired by regression analysis, this work proposed conducting 3-D surface regression by using a neural network. Through the net, the heights of the building scatterers were estimated according to the original scatterer coordinates. Thus, points were refined to the positions along with the ground truth of buildings, and uneven surfaces were smoothed. The experimental results demonstrate that the proposed method can provide fast and precise regularization for urban buildings. It also has excellent denoising performance on raw TomoSAR point clouds. Compared to RANSAC and TomoSeed, our proposed method was more competitive and potential in applications for common polygonal architectures of regular shapes. For irregular buildings with round footprints (e.g., domes and Canton Tower), or fine structures (e.g., stairs), our method still needs to be improved for higher nonlinearity. In the future, we will keep improving our method to fit more complicated city scenes. Moreover, we will try some regularization or increase the complexity of the neural network to maintain detailed information about buildings. Other machine learning approaches will also be taken into consideration.

## Figures and Tables

**Table 1:** Reconstruction precision and time cost obtained by a regular desktop PC (Core i5-7200U). A lower number indicates a better performance. The time does not include the time expended on data splitting and splicing in RANSAC and TomoSeed.

Method	Precision	Time
RANSAC	0.2615 m	40.2 s
TomoSeed	0.1961 m	9 min 38 s
Proposed	0.1784 m	8.77 s

**Table 2:** Reconstruction precision and time cost obtained by a regular desktop PC (Core i5-7200U). A lower number indicates a better performance. The time does not include the time expended on data splitting and splicing in RANSAC and TomoSeed.

Method	Precision	Time
RANSAC	1.7384 m	50.94 s
TomoSeed	1.6420 m	6 min 43 s
Proposed	0.1896 m	24.49 s

**Table 3:** Time cost. Processing times are obtained by a regular desktop PC (Core i5-7200U). The time does not include the time expended on data splitting and splicing in RANSAC and TomoSeed.

Method	Time
RANSAC	48.83 s
TomoSeed	1 min 18 s
Proposed	14.47 s

**Table 4:** Time cost. Processing times are obtained by a regular desktop PC (Core i5-7200U). The time does not include the time expended on data splitting and splicing in RANSAC and TomoSeed.

Method	Time
RANSAC	33.78 s
TomoSeed	18 min 35 s
Proposed	13.94 s
